# High-throughput PacBio library preparation and sequencing techniques for genomic DNA and TNA

**DOI:** 10.3389/fgene.2025.1587691

**Published:** 2025-08-06

**Authors:** Meghan L. Bentz, Cristina Clines, Jasmine Padilla, Christopher Horn, Justin Lee, Mili Sheth

**Affiliations:** ^1^ Genomics Sequencing Laboratory, Centers for Disease Control and Prevention, Atlanta, GA, United States; ^2^ Influenza Division, Centers for Disease Control and Prevention, Atlanta, GA, United States

**Keywords:** WGS whole-genome sequencing, high throughput, PacBio, automation, methods, gDNA, COVID, sequencing

## Abstract

PacBio Sequencing is an effective tool for achieving long read lengths to close gaps in whole genome assemblies. Limited information is available for automated, high-throughput library preparation workflows for PacBio sequencing. This paper describes two high throughput methods, one for producing barcoded genomic DNA libraries and one for barcoded cDNA and subsequent library preparation, as well as pooling and sequencing on the Sequel II instrument. Test sequencing was performed on 380 genomic DNA samples, of which 28 (7.4%) failed sequencing and needed to be repeated. Test sequencing was performed on 384 Threose nucleic acid samples of which 0 failed sequencing and 49 (12.8%) had a genome coverage <90%. These methods were used to generate and report results for ∼2,000 genomic DNA and ∼30,000 cDNA genome sequences from clinical specimens in 2023.

## Introduction

Whole genome sequencing (WGS) and amplicon sequencing are two methods used to study the genomes of organisms. WGS aims to sequence the entire genome and amplicon sequencing focuses on a specific, smaller region of interest. Due to the nature of the target, WGS requires longer fragments of genetic material (referred to as gDNA, or genomic DNA) than amplicon sequencing, and are therefore also referred to as long read vs short read sequencing. There are two platforms that offer long read sequencing in addition to amplicon; Oxford Nanopore and Pacific Biosciences (PacBio) (Schadt et al., 2010; Weirather et al., 2017).

PacBio sequencing employs HiFi (High Fidelity) read technology, which consists of single-molecule, real-time sequencing without pauses between read steps, creating long read lengths (Schadt et al., 2010). These continuous reads can be used to close gaps in assemblies, resolve long repeat regions and mutations, identify gene isoforms, and resolve plasmids in microbial samples (Rhoads and Au, 2015). Additionally, PacBio sequences each base multiple times, leading to improved read accuracy. However, PacBio sequencing is hindered by its lower throughput and high cost point per base when compared to other sequencing platforms. In this project, we attempt to increase the throughput and decrease the cost by applying high throughput methods to PacBio sequencing.

High throughput sequencing is accomplished by preparing many barcoded libraries, often with liquid handlers, and sequence them together in one pool. This cuts down on instrument run time, as all libraries are sequenced simultaneously, but can have problems that a small number of hand prepared barcoded libraries do not experience. Three popular WGS platforms that are adaptable to high throughput methods are Illumina, Oxford Nanopore, and PacBio. Short read Illumina sequencing is a common choice for high throughput and has a very low error rate (i.e. 0.24 ± 0.06% per base (Pfeiffer et al., 2018) but is limited by a maximum read length of 500 bp (2x250 bp paired reads) which prevents collection of long read data and therefore limits resolution of long repeat regions (Johnson et al., 2019). Using the Novaseq v1.5 reagent kit, the Illumina Novaseq 6,000 instrument can sequence up to 40 billion base pairs per run using the two largest output flow cells for which the longest read length is 300 bp (2 x 150 bp paired reads) (Illumina, 2024). Oxford Nanopore Technologies (ONT) and Pacific Biosciences are currently the only two companies manufacturing instruments that can generate long-read sequencing data. ONT instruments have a higher error rate than Illumina and PacBio (i.e. 13.4% per base for ONT 2D (Weirather et al., 2017)) but can sequence molecules up to 2.3 Mb (Payne et al., 2019). Sequencing both strands of a double-stranded template is possible on ONT instruments, and this can increase the accuracy of base-calls relative to sequencing only one strand of each template (Laver et al., 2015). Comparatively, PacBio’s use of circular consensus sequencing (CCS) results in each base in a template being sequenced multiple times followed by consensus basecalling from the resulting subreads. This process generates high quality reads (repeat reads of libraries ∼20 kb and smaller), which combines with the ability to sequence longer read lengths (up to ∼89 kb), resulting in long, accurate scaffolds that yield more complete coverage during whole genome sequencing (Cui et al., 2020) and a low error rate (i.e. 1.72% per base for PacBio CCS (Weirather et al., 2017).

PacBio amplicon sequencing has been previously adapted to a high throughput model via dual-unique molecular identifier (UMI) barcoding (Karst et al., 2021). This is an effective technique for amplicons but is not functional for large genomic DNA (gDNA) as it requires a bespoke primer set targeting a specific smaller region of the genome. It also requires a PCR step that can introduce errors and bias into the final data. Alternatively, hybrid strategies combining reads from two different platforms have been used to improve coverage efficiency. For example, combining PacBio long reads with Illumina short reads (Rhoads and Au, 2015). Unfortunately, this requires proficiency in multiple sequencing platforms and the time, equipment, and reagents for repeat sequencing on multiple platforms.

In this paper we developed methods for adapting lower throughput PacBio sequencing to two high throughput workflows to produce accurate single-molecule consensus sequences. Sequencing is performed with the PacBio Sequel II instrument using 3.0 chemistry, which has an average read length of 10–15 kb with an N50 of more than 50 kb (more than half of the data are in reads >50 kb). There are eight million ZMWs (zero-mode waveguide) on a single SMRT cell, which can produce one subread/CCS read each. The throughput of this system is 0.5-1 billion bases per SMRT cell (Pacific Biosciences, 2024a).The first high throughput method we describe in this paper is specific to gDNA and the second method is for targeted Threose Nucleic Acid (TNA) sequencing. Validation studies performed on clinical specimens showed robust WGS coverage in a multiplexed setting. Using these methods, we successfully converted our lower-throughput methodology to an automated, high-throughput process. These methods have been used to report results for ∼2,000 (gDNA) and ∼30,000 (cDNA) genome sequences from clinical specimens in 2023.

## Materials and equipment

### Reagent kits

HiFiViral SARS-CoV-2 target capture kit (Molecular loop, ML5200-PB).

Barcoded M13 primer plate (Molecular loop, ML2200-PB-384).

SMRTbell adapter index plate 96A (PacBio, 102-009-200).

SMRTbell prep kit 3.0 (PacBio, 102-182-700).

Sequel Binding kit 3.1 (PacBio, 102-333-400).

Sequel Binding kit 3.2 (PacBio, 102-333-300).

gDNA 165 kb Femto size Analysis kit (275 samples) (Agilent, FP-1002-0275).

Blue Pippin 0.75% 1–10 kb kit (Sage Science, BLF7510).

### Plastics

Hardshell 384-well PCR plate (Revvity, 6008910).

Hardshell 96-well PCR plate (Revvity, 6008870).

Optically clear, adhesive seal pack of 100 (Biorad, MSB1001).

### Equipment

Sequel II sequencer (PacBio).

Mosquito liquid handler (SPT, 4150-03033).

Spool of 18500 gamma ray sterilised pipettes 4.5 mm pitch HV (for 384+ well plates) (SPT, 4150-03033).

Spool of 13500 gamma ray sterilised pipettes 9 mm pitch HV (for 96 well plates) (SPT, 4150-03032).

Zephyr liquid handler (Revvity, 111624).

Caliper 80uL Barrier 96 SBS racked Pippette tips pre-sterilized (Revvity, 111624).

Femto Pulse (Agilent).

Blue Pippin (Sage Science).

## Methods

Two high-throughput PacBio sequencing workflows were developed and tested on prepared samples. The first workflow describes the rapid, high throughput preparation of 96 gDNA libraries and subsequent sequencing. The second workflow describes the high throughput preparation of 384 barcoded cDNA from TNA input and subsequent library preparation and sequencing. All test samples were sequenced using SMRTlink version 11.

### Samples

TNA and gDNA samples were extracted then evaluated for quality using Nanodrop, Qubit, and Fragment Analyzer/Femto Fragment Analyzer.

The extractions for gDNA were performed on *E. coli* positive clinical specimens in the Global Health Kenya laboratory at Washington State University, Pullman, Washington, United States. The extraction kit used was the Qiagen-MagAttract HMW DNA kit (Qiagen, 67563).

The extractions for TNA were performed on SARS-CoV-2 positive clinical specimen (Ct 12-33) in the Core Operation and Outbreak Response laboratory at the U.S. Centers for Disease Control and Prevention (CDC), Atlanta, Georgia, United States. Using a 96 well MP96 processing cartridge, 100ul of each specimen was added to 350ul of MP96 External Lysis Buffer and incubated at room temperature for 10 min for virus inactivation. After the incubation the cartridge was loaded onto a MagNA Pure 96 (MP96) extraction platform and TNA was extracted using the MagNA Pure 96 DNA and Viral NA SV Kit (Roche, 06543588001).

### High throughput gDNA library preparation and sequencing

Libraries were prepared using the SMRTbell prep kit 3.0 (PacBio, 102-182-700) and the SMRTbell barcoded adapter plate 3.0 (PacBio, 102-009-200). The liquid handlers used for this method include the HV Mosquito (SPT Labtech, 4150-03032, HV 9 mm pitch tips) and the Zephyr (Revvity 111624, Pipette Filter Tips, 80 µL). Twelve hand prepped gDNA libraries were used as a control for comparison with the high throughput library preparation.

High throughput adjustments were made to PacBio protocol “Preparing whole genome and metagenome libraries using SMRTbell prep kit 3.0” (Pacific Biosciences, 2024b). Library preparation reactions were reduced to 1/3 volume. Master mixes were prepared on ice in 1.5 mL lo-bind tubes, mixed, and spun down. When preparing the 384-well reagent plate (Revvity, HardShell PCR Plate, 384-well 6008910), the pipette tip was washed 3x in master mix before dispensing into the reagent plate. The multiple washes coat the interior of the tip with reagent, allowing for accurate volume dispersion without the need for a second stop release which would add air bubbles to the reagent well. Sample quality was determined via Qubit (1x dsDNA HS assay, ThermoFisher, Q33231) and Femto Pulse (gDNA 165 kb analysis kit, Agilent, FP-1002-0275) size analysis before library prep, after library prep, and after size selection.

**FIGURE 1:**
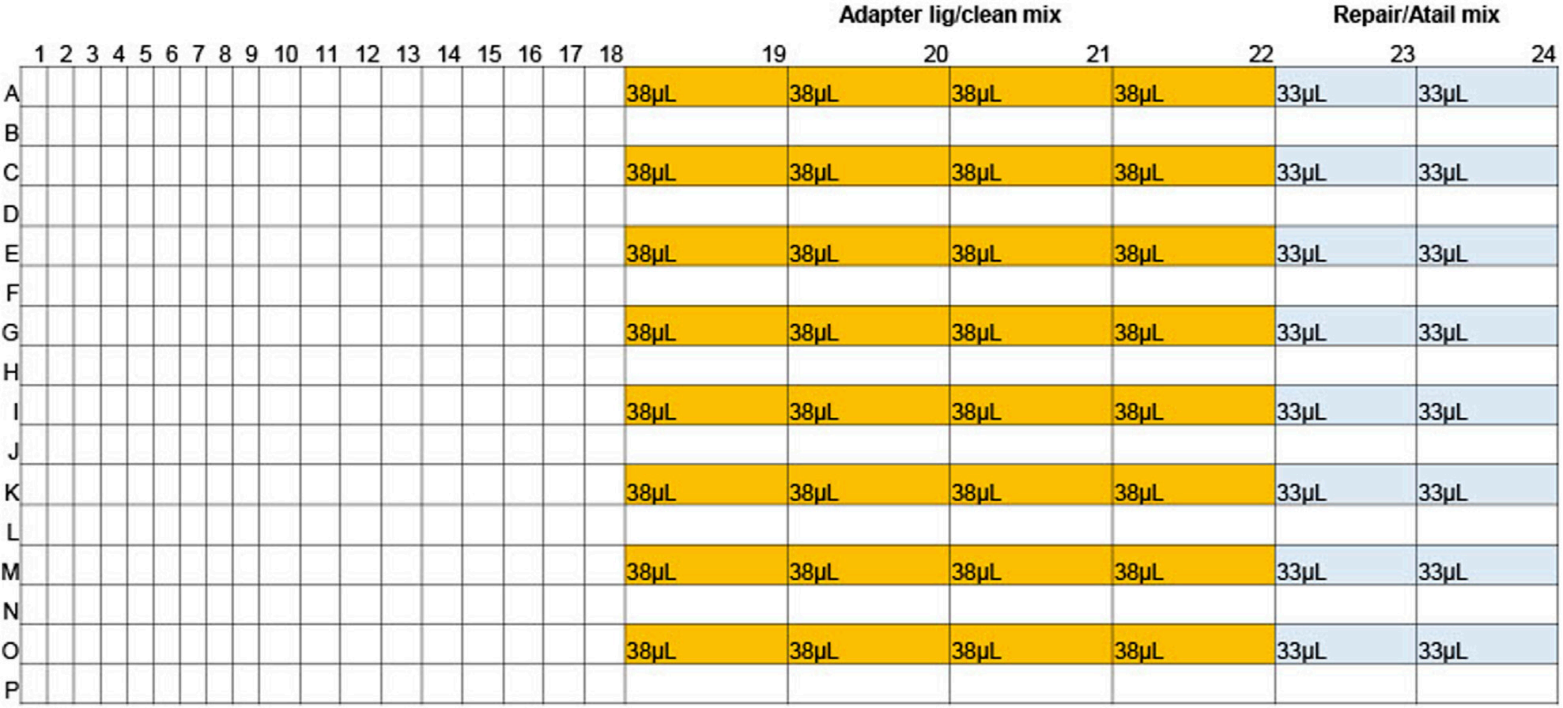
Reagent plate layout for gDNA library preparation.

For the first step, 15.3 µL of gDNA was dispensed into a 96-well plate (Revvity, HardShell PCR Plate, 96-well 6008870), hereafter called the gDNA reaction plate. The plate was sealed with Corning aluminium sealing foil (Thermofisher, 07-200-684), spun down, and kept on ice until use. 532µL Repair/Atail master mix (Pacific Biosciences, 2024b) was prepared and dispensed into two columns of a 384-well plate (Revvity, 6008910), hereafter called the gDNA reagent plate (Figure 1). The gDNA reagent plate was sealed with aluminium sealing foil, spun down, and kept on ice until use. gDNA reagent and reaction plates were both unsealed, then using the Mosquito equipped with HV 9 mm pitch tips, 4.67 µL Repair/Atail master mix was added to each sample well in the reaction plate. The gDNA reagent plate was then sealed with a foil seal and set on ice. The gDNA reaction plate was sealed tightly with an optically clear adhesive seal (Biorad, MSB1001) and mixed by inverting and tapping the plate on the palm of the hand two times on each face of the plate. This was repeated three times to ensure thorough mixing. The plate was then spun down and placed in the thermal cycler for the Repair & A-tailing reaction (Pacific Biosciences, 2024b).

**FIGURE 2:**
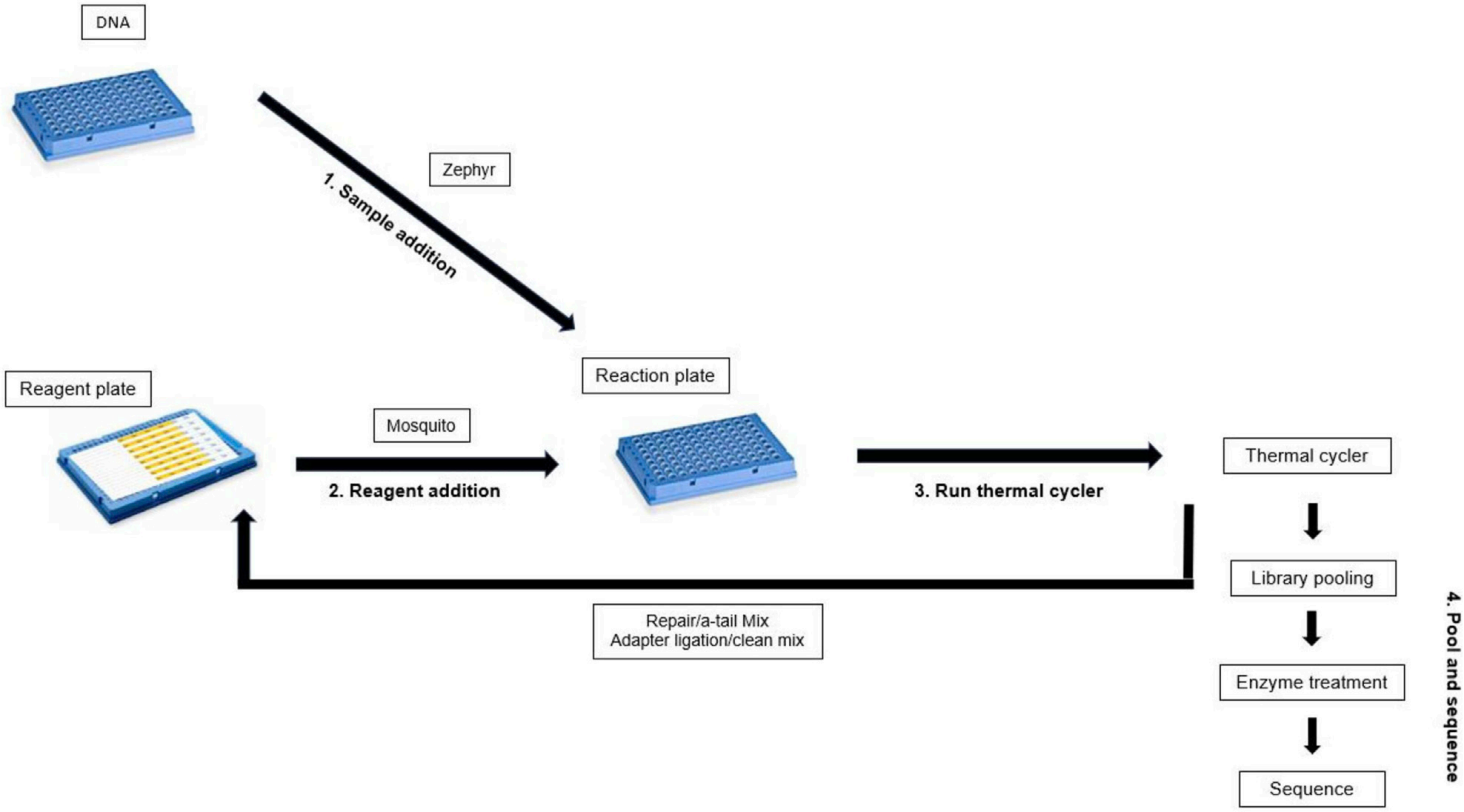
Workflow for gDNA library preparation.

For the second reagent addition, the SMRTbell barcoded adapter plate 3.0 (PacBio, 102-009-200) was thawed and spun down, then kept on ice until use. 1,220 µL Adapter ligation/Clean master mix (Pacific Biosciences, 2024b) was prepared and dispensed into 32 wells in columns 19-22 of the gDNA reagent plate (Figure 1). The gDNA reagent plate was then sealed with aluminium sealing foil, spun down, and kept on ice until use. Once the Repair/A-tailing thermal cycler program was complete, the gDNA reaction plate was thoroughly spun down and kept on ice until use. The SMRTbell barcoded adapter plate 3.0 and reaction plate were then unsealed, and the Mosquito was used to add 1.3 µL barcode to the gDNA reaction plate. The barcode plate was discarded after use and the gDNA reagent plate unsealed and placed on the Mosquito. The Mosquito was then used to add 10.3 µL Adapter ligation/clean mix to the gDNA reaction plate. The gDNA reagent plate was discarded, and the gDNA reaction plate was sealed tightly with an optically clear adhesive seal and mixed by inverting and tapping the plate on the palm of the hand two times on each face of the plate. This was repeated three times to ensure thorough mixing. The plate was then spun down and placed in the thermal cycler for the Adapter ligation/clean program (Pacific Biosciences, 2024b) (Figure 2).

To determine how many samples should be pooled together we used the PacBio recommended maximum loading of 375 Mb (Pacific Biosciences, 2022b) divided by the estimated genome size of the organisms being sequenced. When pooling we kept organisms with similar genome sizes and library sizes together in the same pool. An equivolume pool was then created using the Mosquito to pool 5µL/sample into two columns of a 384 plate which were then pooled into two tubes. Each tube underwent a 2X bead clean using Ampure PB beads (PacBio, 100-265-900) and was eluted into 40 µL elution buffer (PacBio, 101-633-500). Each tube then underwent nuclease treatment (Pacific Biosciences, 2024b) then another 2X bead clean using Ampure PB beads before being eluted into 50 µL elution buffer. The Blue Pippin was then used to size select for >10 kb libraries using the Blue Pippin 0.75% 1–10 kb kit (Sage Science, BLF7510). The Blue Pippin output underwent a 2x bead clean using Ampure PB beads and was eluted into a volume of 60 µL elution buffer.

The final size selected library pool was prepared for sequencing using the Sequel Binding kit 3.2 (PacBio, 102-333-300). Binding kit conditions were determined by SMRTLink Sample Setup with two modifications included to increase the number of successful reads. Firstly, 2X the recommended polymerase was used for the polymerase binding step and secondly the library was loaded at 34% higher than the recommended concentration. The library pools were sequenced on the Sequel II instrument.

PacBio HiFi long reads were assembled using CLC Genomics Workbench with default parameters utilizing the inbuilt hifism assembler tool (Qiagen, 2025; Cheng et al., 2021). A minimum contig length of 10,000 bp was set, with the contigs kept circular. Genome size was inferred automatically by the assembler based on the sequences being processed. Assembled genomes with an N50>4.5 were considered successfully assembled, while those below the threshold were excluded from the analysis. Expected genome size for *E. coli* and *Pneumonia* samples was ∼5 Mb which informed the set N50 threshold.

### High throughput cDNA preparation and sequencing

Before library preparation TNA samples were converted into cDNA using the Molecular Loop HiFiViral SARS-CoV-2 target capture kit (Molecular loop, ML5200-PB) and barcoded using the Barcoded M13 primer plate (Molecular loop, ML2200-PB-384). The reagent ratios are included in the Molecular Loop protocol “Viral RNA Target Capture Kit PacBio (combined cleanup)” (Molecular Loop Biosciences, 2022). The liquid handlers used for this method include the Mosquito (SPT 4150-03033, HV 4.5 mm pitch tips) and the Zephyr (Revvity 111624, Pipette Filter Tips, 80 µL). Twelve hand prepped cDNA samples were used as a control for comparison with the high throughput cDNA preparation.

**FIGURE 3:**
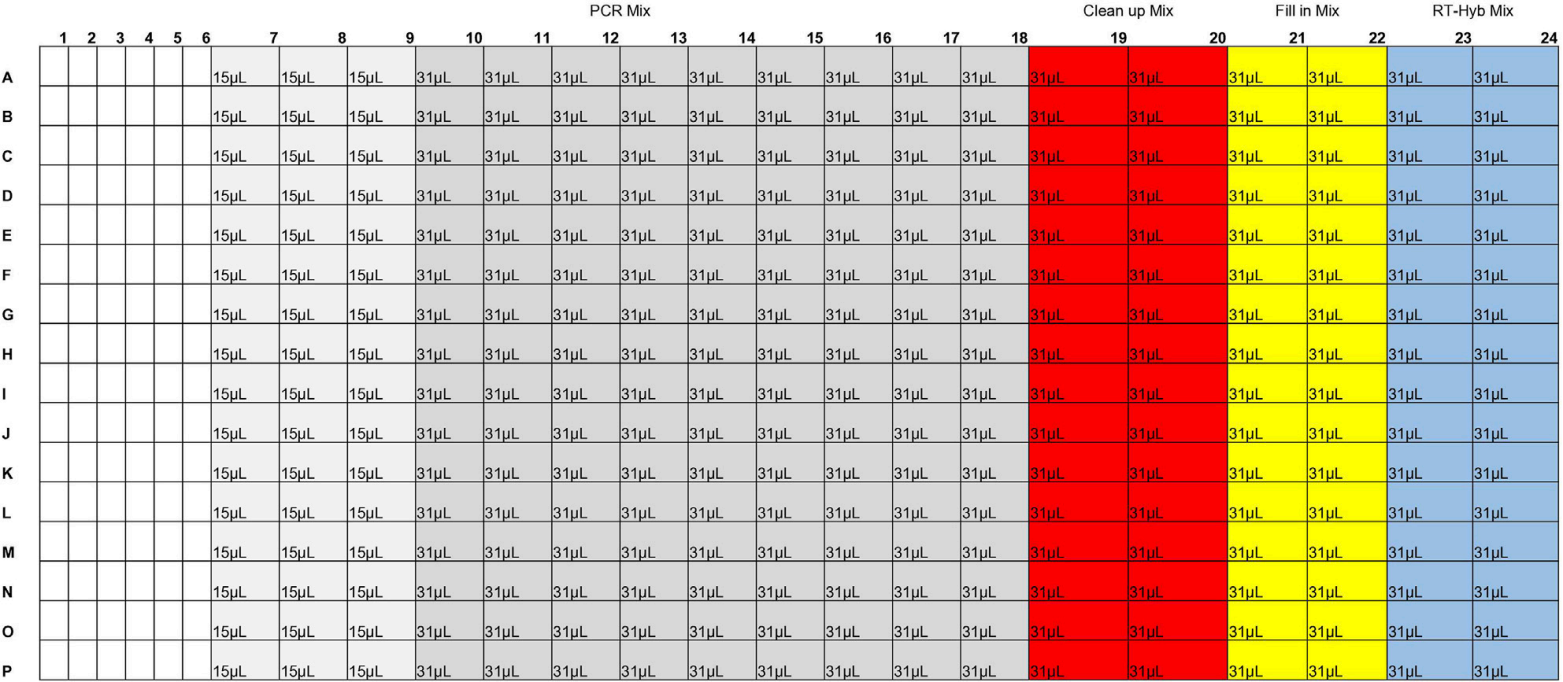
Reagent plate layout for TNA cDNA preparation.

Master mixes were prepared on ice in 1.5 mL lo-bind tubes, mixed, and spun down. When preparing the 384-well reagent plate (Revvity, HardShell PCR Plate, 384-well 6008910), the pipette tip was washed 3x in master mix before dispensing into the reagent plate. A Zephyr was used to add 6 µL sample to each well of a 384-well plate, hereafter referred to as the cDNA reaction plate. The cDNA reaction plate was then sealed with foil, spun down, and kept on ice until use. 992µL of RT-Hybridization master mix was dispersed into two columns of a 384-well plate (Figure 3), hereafter called the cDNA reagent plate. The cDNA reagent plate was then sealed with foil, spun down, and kept on ice until use. A Mosquito equipped with HV 4.5 mm pitch tips was then used to dispense 2 µL RT-Hybridization master mix into each well of the cDNA reaction plate. The cDNA reagent plate was sealed with a foil seal and set on ice. The cDNA reaction plate was then sealed tightly with an optically clear adhesive seal and mixed by vortexing thoroughly. The cDNA reaction plate was spun down and placed in the thermal cycler for the RT-Hybridization program (Pacific Biosciences, 2022a).

Fill-in master mix was dispersed into two columns of the cDNA reagent plate (Figure 3). The plate was then sealed with foil, spun down, and kept on ice until use. The cDNA reaction plate was removed from the thermal cycler, spun down, and kept on ice until use. A Mosquito was used to dispense 2 µL Fill-in mix into each well of the cDNA reaction plate. The cDNA reagent plate was sealed with a foil seal and set on ice. The cDNA reaction plate was then sealed tightly with an optically clear adhesive seal and mixed by vortexing thoroughly. The cDNA reaction plate was spun down and placed in the thermal cycler for the Fill-in reaction (Pacific Biosciences, 2022a).

Clean-up master mix was dispersed into two columns of the cDNA reagent plate (Figure 3). The cDNA reagent plate was then sealed with foil, spun down, and kept on ice until use. The cDNA reaction plate was removed from the thermal cycler, spun down, and kept on ice until use. A Mosquito was used to dispense 2 µL Clean-up mix into each well of the cDNA reaction plate. The cDNA reagent plate was sealed with a foil seal and set on ice. The cDNA reaction plate was then sealed tightly with an optically clear adhesive seal and mixed by vortexing thoroughly. The cDNA reaction plate was then spun down and placed in the thermal cycler for the Clean-up reaction (Pacific Biosciences, 2022a).

PCR master mix was dispersed into 12 columns of the cDNA reagent plate (Figure 3). The cDNA reagent plate was then sealed with foil, spun down, and kept on ice until use. The M13 barcode plate was thawed, spun down, and kept on ice until use. The cDNA reaction plate was removed from the thermal cycler, spun down, and kept on ice until use. A Mosquito was used to dispense 2.4 µL of barcode from the M13 barcode plate to the cDNA reaction plate. A Mosquito was used to dispense 12 µL PCR mix into each well of the cDNA reaction plate. The cDNA reagent plate and M13 barcode plate were then discarded, and the cDNA reaction plate was sealed tightly with an optically clear adhesive seal and mixed by vortexing thoroughly. The cDNA reaction plate was spun down and placed in the thermal cycler for the cDNA amplification reaction (Pacific Biosciences, 2022a).

**FIGURE 4:**
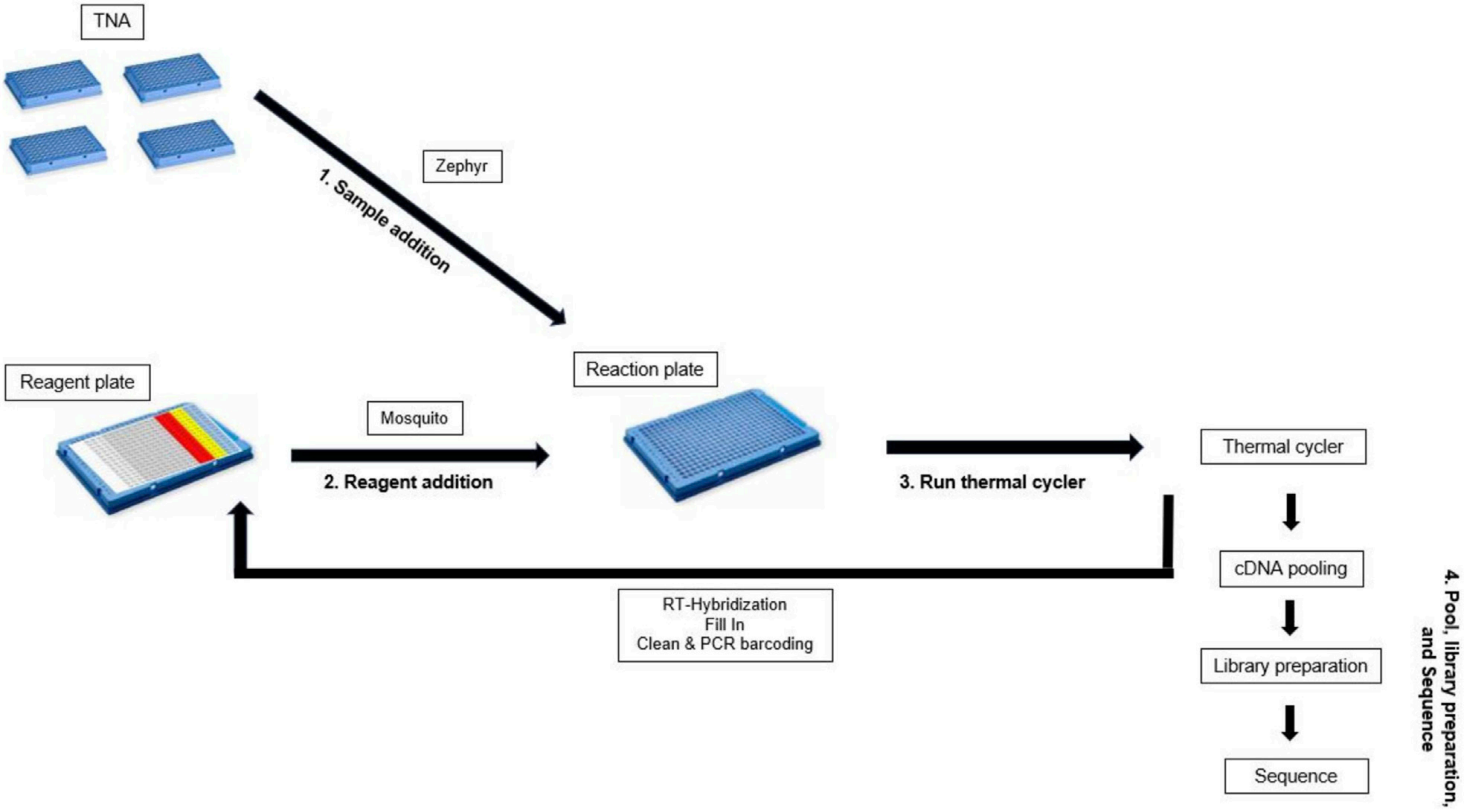
Workflow for TNA cDNA preparation.

Once cDNA was prepared an equivolume pool was created with 5 µL of each sample. A 1.8X bead clean was then performed using Promega ProNex Size-Selective Purification beads, eluting into 50 µL PacBio elution buffer. Libraries were prepared using the SMRTbell prep kit 3.0 (PacBio, 102-182-700) with <1000 ng input from the equivolume sample pool (Figure 4).

The final library pool was prepared for sequencing using the Sequel Binding kit 3.1 (PacBio, 102-333-400). Binding kit conditions were determined by SMRTLink Sample Setup with one modification. 0.5X the recommended polymerase was used for the polymerase binding step. The library pools were sequenced on the Sequel II instrument.

## Results

### High throughput gDNA library preparation and sequencing

The first method allows for the rapid preparation of 96 high quality gDNA libraries in ∼2hrs and 30 min. The process was miniaturized, with the total volumes reduced to 1/3 what was described in the protocol “Preparing whole genome and metagenome libraries using SMRTbell prep kit 3.0” (Pacific Biosciences, 2024b). A Mosquito instrument (SPT Labtech) was used to transfer master mix from a 384-well reagent plate (Figure 1) to a 96-well reaction plate containing samples. Initial tests with the Mosquito instrument showed that a 96-well plate required a minimum starting volume higher than a 384-well plate, leading to greater reagent overage needs. Because of this, a 384-well plate was used as the reagent plate. A 96-well plate was used as the reaction plate as the larger capacity was more effective for the manual mixing method, which provided better formed libraries. The 14.6 µL gDNA added initially to the reaction plate was a sufficient volume to overcome the minimum starting volume requirements of the 96-well plate. To minimize reagent overage the least possible number of reagent wells were used. The 384-plate wells have a maximum capacity of ∼40µL, so the reagent master mixes were divided into multiple wells to account for the low capacity. 17.8% overage was needed per reagent well for Repair/Atail master mix (1.11% per sample) and 23% overage was needed per reagent well for Adapter/ligation master mix (0.7% per sample). After reagent addition the plates were manually mixed instead of vortexing or pipetting to avoid fragmenting the DNA/libraries.

After library preparation the gDNA samples were pooled before undergoing enzyme treatment to remove malformed SMRTbells (Pacific Biosciences, 2024b) and underwent size selection to remove SMRTbells under 10 kb. Pooling was based on PacBio recommended maximum loading of 375 Mb for the Sequel II instrument (Pacific Biosciences, 2022b). Post-run statistics met PacBio recommended run criteria (Pacific Biosciences, 2020).

Adjustments to the loading conditions provided by SMRTLink were tested using an equivolume pool of 96 libraries from bacterial isolate gDNA with an estimated average genome size of 5 Mb.

**FIGURE 5:**
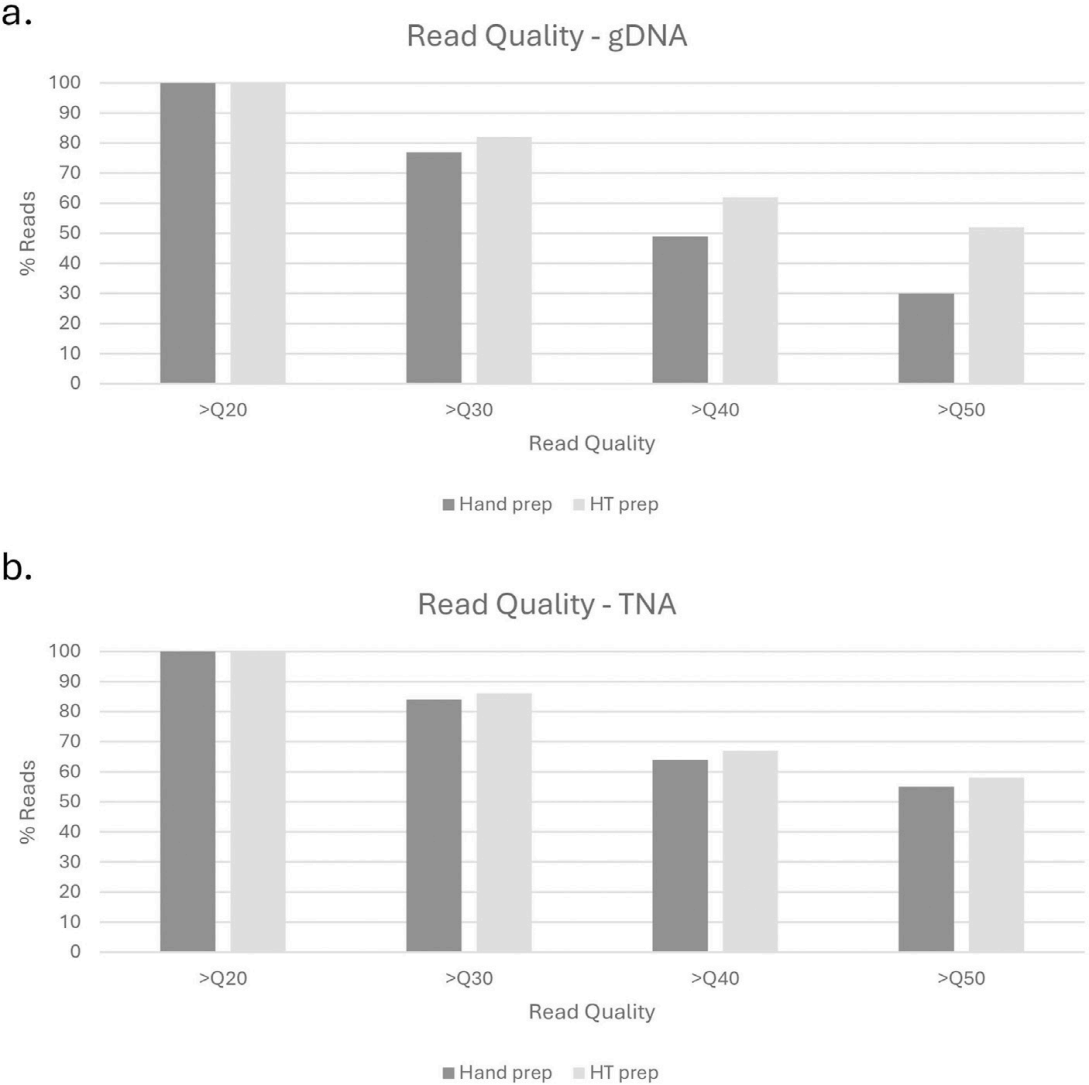
Read quality comparison of hand prep vs. High Throughput prep for gDNA **(a)** and TNA **(b)** methods.

Test library preparation and sequencing was performed on 380 gDNA samples using the described high throughput method. The estimated genome size was 5Mb, with samples prepared in an equivolume (5µL/sample) pool of 72 samples for sequencing. When compared to the 12 hand prep gDNA library controls, the high throughput pep had fewer unbarcoded reads (5.38% as compared to 10.77% for hand prep), a similar polymerase read length (63.6 kb as compared to 76.68 kb for hand prep), and a slight increase in read quality (Q53 as compared to Q40 for hand prep) (Figure 5).

### High throughput cDNA preparation and sequencing

The second high throughput method allows for the rapid preparation of 384 barcoded cDNAs. Reagent volumes are described in the protocol “Viral RNA Target Capture Kit PacBio (combined cleanup)” (Molecular Loop Biosciences, 2022). A Mosquito instrument was used to add reagents from a prepared 384-well reagent plate (Figure 3) to a 384-well reaction plate containing TNA sample and the plates were sealed with optically clear adhesive seals before vortexing. Given that mixing occurred via vortexing rather than manually, a 384-well plate was used rather than a 96-well plate as the small well size of the 384-well plate adhered better to the clear adhesive seals and required less reagent overage than the 96-well plate. When testing in a 96-well plate the plastic plate the seals did not attach as strongly and sometimes detached during the RT-hybridization step, which led to an increased risk of contamination and reagent evaporation. To minimize reagent overage needs the reagents were placed in the minimum number of wells possible in the reagent plate. RT-Hybridization, fill in, and cleanup master mixes required 29% overage per reagent well (2.4% per sample). Given the upper limit of 5uL per tip for the Mosquito 4.5 HV tips, 4uL of PCR master mix was transferred three times to the reaction plate to achieve the final volume addition of 12uL. Each 31uL well was drawn from seven times (requiring 10.7% overage) and each 15uL well was drawn from three times (requiring 25% overage). Overall, the PCR master mix required 12.5% overage per sample. Reagent plates were sealed and protected with foil seals, as the clear adhesive seals would charge the reagent plate wells with static, leading to increased sample loss. The Molecular Loop Barcoded M13 Primer Plate was used for barcoding as it was compatible with the combined cleanup-PCR step. Miniaturization of this method was attempted at half and third volumes, but the barcoded cDNA failed to form under these conditions (evaluated via qubit and Fragment Analyzer size analyzer, with no signal of target cDNA size). After the barcoded cDNA was prepared, the samples were pooled in an equivolume pool before undergoing library preparation and enzyme treatment to remove malformed smrtbells, and sequencing.

Adjustments to loading conditions were tested using an equivolume pool of 384 cDNA with an average length of 800bp.

Test sequencing was performed on 384 TNA samples using the described high throughput method. The estimated genome size was 29kb, with samples prepared in an equivolume (5µL/sample) pool of 384 samples for library preparation and sequencing. When compared to the 12 hand prep cDNA controls, the high throughput pep had fewer unbarcoded reads (3.52% as compared to 11.45% for hand prep), the same polymerase read length (0.8 kb), and a slight increase in read quality (Q65 as compared to Q59 for hand prep) (Figure 5).

## Discussion

### High throughput gDNA library preparation and sequencing

**FIGURE 6:**
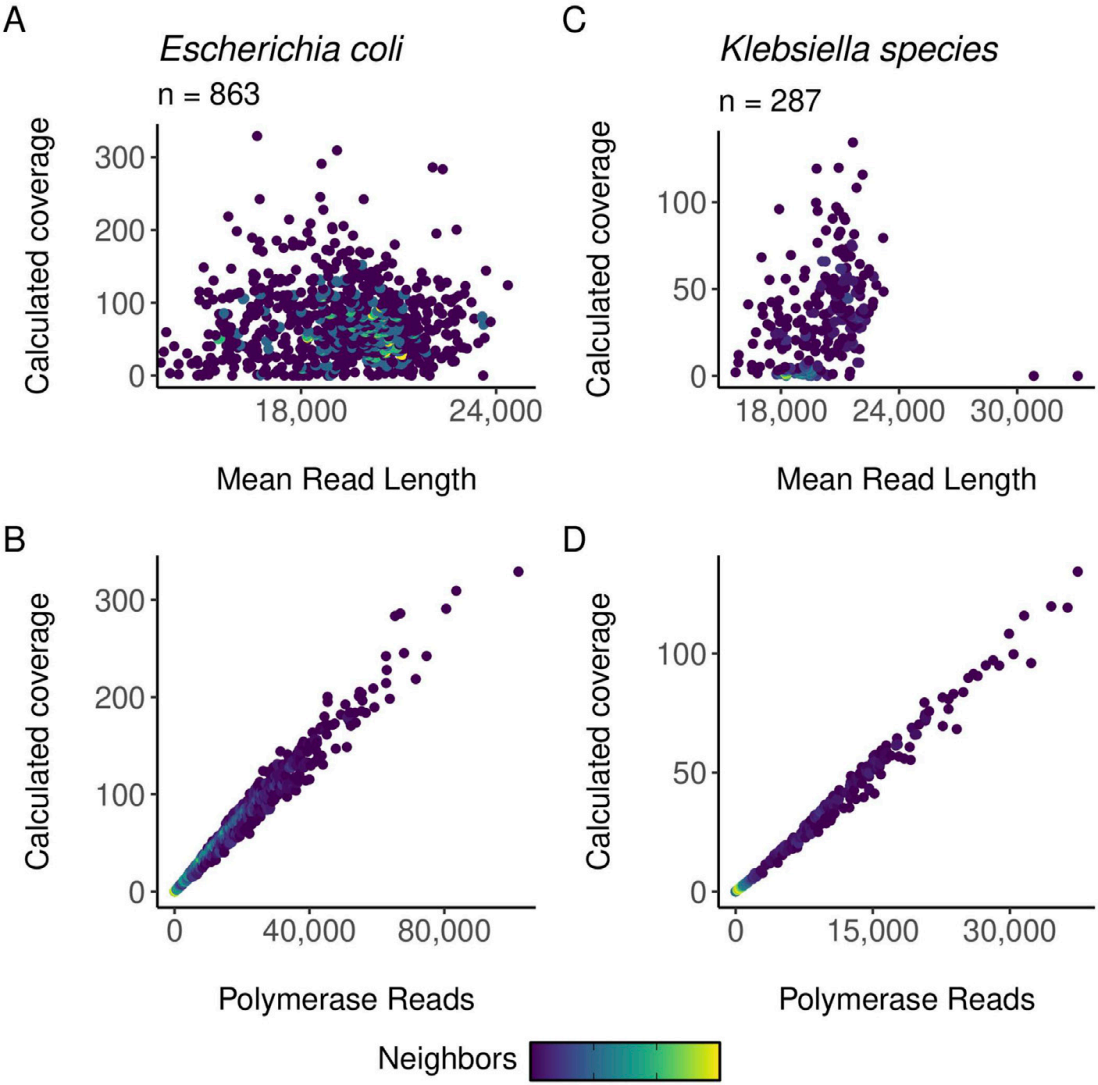
gDNA calculated coverage compared to polymerase reads and mean read length. In all plots, point density/neighbors are mapped to color, with light colors indicating more dense areas of data. Coverage is calculated as bases/genome size. The points are color-mapped based on the number of neighboring points using the ggpointdensity R package. **(A)** Scatter plots of calculated coverage against mean read length for E. coli samples. **(B)** Scatter plots of calculated coverage against mean polymerase reads for E. coli samples. **(C)** Scatter plots of calculated coverage against mean read length for Klebsiella samples. **(D)** Scatter plots of calculated coverage against mean polymerase reads for Klebsiella samples.

**FIGURE 7:**
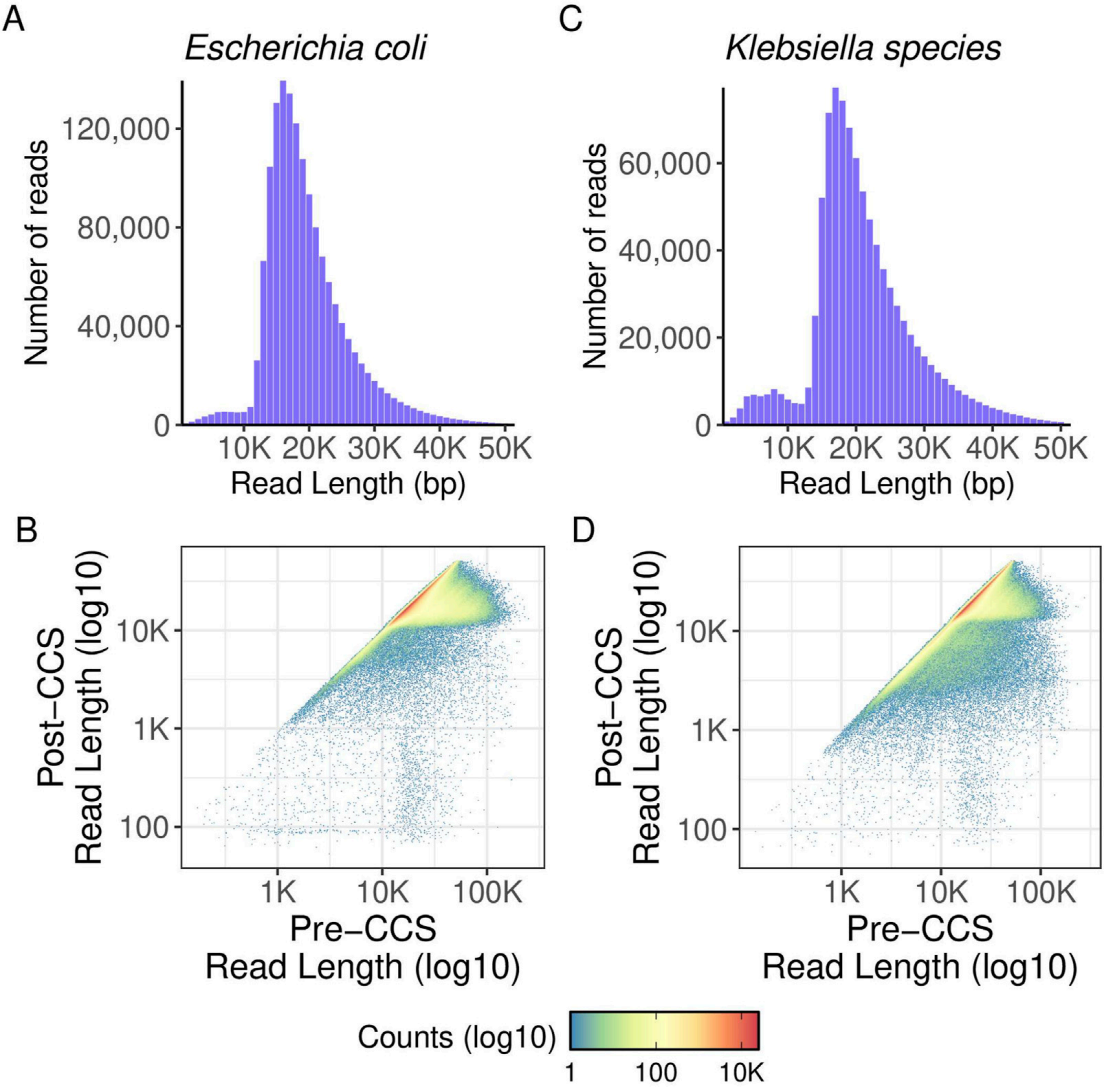
gDNA read length density and polymerase read length. Histograms showing read length distribution of aggregated data. Pre-CCS read lengths were found by finding the maximum read length of a given ZMW prior to matching it to its corresponding post-CCS read length. **(A)** read length density for E. coli samples. **(B)** polymerase read length distribution for E. coli samples. Pre- and post-CCS read lengths are shown. **(C)** read length density for Pneumococcus samples. **(D)** polymerase read length distribution for Pneumococcus samples. Pre- and post-CCS read lengths are shown.

Preliminary and post-run analyses in SMRTLink had comparable results for the high throughput method described in this manuscript and the standard manual method of gDNA library preparation. Figure 6 shows the distribution of mean read lengths and polymerase read numbers across the gDNA HT prep library pools. The polymerase is seen to behave as expected with more polymerase reads improving calculated coverage and a wide distribution in mean read length. Figure 7 shows the variation in number of gDNA reads as read length increases. A peak can be seen around the expected size of 20 kb with minimal small reads. This indicates that the high throughput method has little to no negative effect on read length size, and no accidental shearing occurs which could potentially interfere with length of reads. When comparing read quality, the high throughput method shows a slight increase in quality as compared to the hand prep method (Figure 5).

When testing adjustments to loading conditions using an equivolume pool of 96 bacterial gDNA libraries, doubling the polymerase concentration resulted in a decrease in the %P0 value and an increase in the %P1 value (Table 1), indicating that fewer SMRTbells had no polymerase attachment (failed sequencing) and more SMRTbells were successfully read by a single attached polymerase. The effects of this change in %P values were seen as an increase in the mean read length and a slight decrease in the number of barcoded reads and maximum reads. Increasing the loading concentration by 34% and 44% resulted in a decrease in the %P0 value, an increase in the %P1 value, and a slight increase in the %P2 value (Table 1), indicating improved sequencing success. The effects of the increase in the %P1 value were seen in an increase in the number of barcoded reads and maximum reads. The mean read length decreased minimally but was still within PacBio recommended performance expectations and could be due to sample pool variability. Though a 44% increase in loading concentration led to a higher number of barcoded reads, for the samples described in this paper we used a 34% increase in loading concentration to decrease the chance of overloading. Since concluding this testing of loading conditions, we have altered our laboratory workflows to increase the loading concentration by 56% for gDNA pools, which has increased the number of successful reads while not leading to noticeable interference from overloading.

**TABLE 1:** Sequel II testing of loading conditions for gDNA.

Conditions	Loading concentration	%P0	%P1	%P2	Barcoded reads	Max reads	Mean read length
CONTROL (1x polymerase, SMRTlink recommended loading concentration)	90pM	64.7	34.4	0.9	557,298	18,087	20,791
2x polymerase	90pM	61.5	37.6	0.9	527,652	16,909	21,249
+34% loading concentration	120pM	56.3	42.3	1.4	651,881	21,181	20,015
+44% loading concentration	130pM	42.7	55.5	1.8	753,074	24,277	20,235

When testing this library preparation method on 380 bacterial gDNA samples, 28 (7.4%) failed sequencing and needed to be repeated. 375 of the 380 total gDNA samples produced closed contigs (Table 2). 267 of the 375 analysed genomes produced circularized contigs. 371 of the 375 analysed samples matched the target species.

**TABLE 2:** Number of gDNA samples producing closed contigs.

Number of closed contigs	Number of samples
0	5
1	18
2	81
3	132
4	78
5	35
6	20
7	3
8	2
9	1
10	2
17	1
34	1
55	1

Total = 380 Samples.

### High throughput cDNA preparation and sequencing

When testing adjustments to loading conditions using an equivolume pool of 384 viral cDNA libraries, halving the polymerase concentration resulted in an increase in %P0, an increase in %P1, and a significant decrease in %P2 (Table 3), indicating that more SMRTbells were successfully read by a single attached polymerase. An elevated %P2 means that the SMRTbell libraries have multiple polymerases attached, which can then interfere with each other in producing successful long reads. The significant decrease in %P2 under test conditions indicates an improved run quality. This improvement in %P1 and decrease of %P2 led to an increase in the number of barcoded reads, maximum reads, and mean read length (Table 3).

**TABLE 3:** Sequel II testing of loading conditions for TNA.

Conditions	Loading concentration	%P0	%P1	%P2	Barcoded reads	Max reads	Mean read length
CONTROL (1x polymerase)	300pM	0.7	63.7	36	3,140,275	24,729	722
0.5x polymerase	300pM	14	76.9	9.1	3,846,208	29,696	729

**FIGURE 8:**
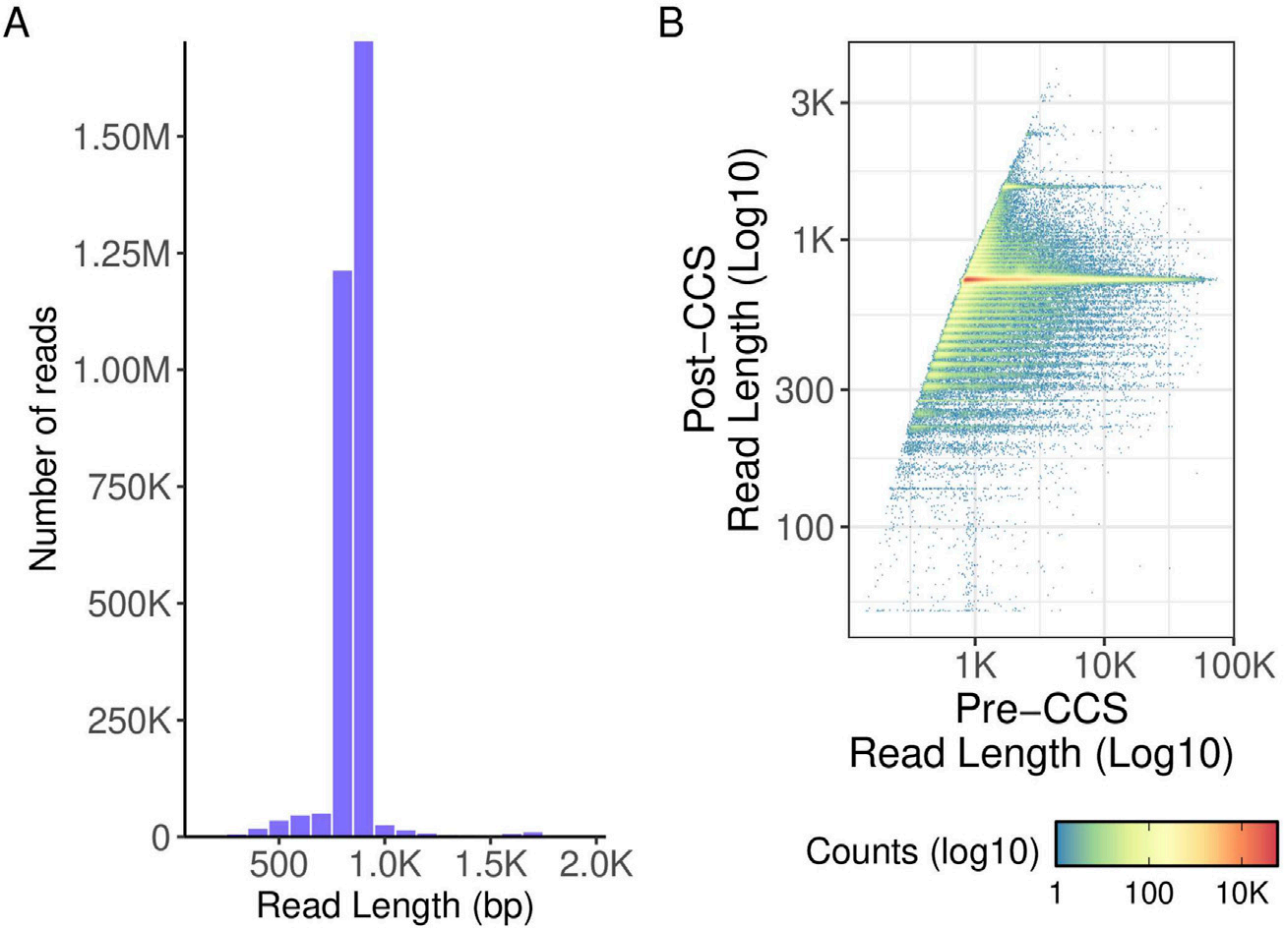
TNA read length density and polymerase read length. **(A)** histogram of read lengths. A cutoff (read length ≤2000 base pairs) was applied to highlight the data. Approximately 0.1% (3009) reads) were thus excluded from the final plot. **(B)** Pre- and post-CCS read lengths. Pre-CCS read lengths were found by finding the maximum read length of a given ZMW prior to matching it to its corresponding post-CCS read length.

When testing this library preparation method on 384 viral TNA samples, 0 failed sequencing and 49 (12.8%) yielded a genome coverage <90%. The read lengths were as expected with a peak at around 800bp (Figure 8). When comparing read quality, the high throughput method shows a slight increase in quality as compared to the hand prep method (Figure 5).

A cost comparison analysis was completed, comparing the PacBio/Molecular Loop high throughput TNA sequencing with Illumina/IDT high throughput TNA MiSeq sequencing workflow. This comparison found the pricing to be very similar per sample, with the PacBio/Molecular Loop costs ∼2.5% lower than Illumina/IDT when considering reagent overages necessary for automation.

Throughput is a limitation to consider for these workflows due to the restricted number of WGS samples that can be run on the Sequel II system. For Microbial multiplexing a single Sequel II SMRTcell has a maximum loading capacity of 375 Mb, and sequencing many samples in one pool can lead to overloading and failed sequencing. This is more likely to happen in organisms with very large genomes. This can be remedied by decreasing the number of samples in a pool, but this will increase the per-sample cost. In future projects we plan to evaluate the higher throughput capabilities of PacBio’s new sequencer, the Vega, The Vega offers an output of 60Gb, which is significantly greater than the 10–15 Gb provided by the Sequel II system. Another limiting factor of PacBio sequencing is the high price point of reagents and quick expiration dates. High throughput can reduce the sequencing cost per sample, but this relies on having a stock of more than 96 WGS samples available with reagent’s minimum expiration window of over 90 days.

To summarize, with PacBio’s continued improvement in read capacity and decreased error rates, it is a strong option for WGS users. While the platform can sequence amplicons, its strength lies in its long read technology, which holds significant potential for analysing organism genomes and studying microbial communities. With these two high throughput workflows available, there is potential to multiplex PacBio sequencing to lower the cost per sample and produce a high number of quality libraries that can be sequenced quickly and efficiently.

## Data Availability

The datasets presented in this article are not readily available because the samples were obtained from a third party and the data has not been published through them yet. Requests to access the datasets should be directed to Kara Moser (qsy5@cdc.gov) and Douglas Ruben (drcall@wsu.edu).
